# Supplementary material for: Analysis of ancestry-specific polygenic risk score and diet composition in type 2 diabetes
Source: PLoS One. 2023 May 23;18(5):e0285827. doi: 10.1371/journal.pone.0285827 (PMC10204962; doi:10.1371/journal.pone.0285827)
Supplement: S1 Table — Abbreviations: dbGaP, Database of Genotypes and Phenotypes; ARIC, Atherosclerosis Risk in Communities study [16]; CARDIA, Coronary Artery Risk Development in Young Adults Study [17]; CHS, Cardiovascular Heart Study [18]; FHS, Framingham Heart Study Offspring and GENX 3 studies [19]; MESA, Multi-Ethnic Study of Atherosclerosis Study [20]; WHI, Women’s Health Initiative study [21]. (DOCX) [file pone.0285827.s001.docx]

**S1. Table. Datasets utilized in our study from** **dbGaP.**

| **Datasets** | **Accession Numbers** |
| --- | --- |
| ARIC | phg000035, phs000280 |
| CARDIA | phg000098, phs000285 |
| CHS | phg000077, phs000287 |
| FHS OFFSPRING | phg000006, phs000007 |
| FHS GENX3 | phg000006, phs000007 |
| MESA | phg000071, phs000209 |
| WHI | phg0000398, phg0000148, phs000200 |
