# Supplementary material for: Analysis of ancestry-specific polygenic risk score and diet composition in type 2 diabetes
Source: PLoS One. 2023 May 23;18(5):e0285827. doi: 10.1371/journal.pone.0285827 (PMC10204962; doi:10.1371/journal.pone.0285827)
Supplement: S2 Table — (DOCX) [file pone.0285827.s002.docx]

**S2 Table. Form of covariates used from all NHLBI Care datasets.**

| **Covariates** | **Form in Model** |
| --- | --- |
| Age | continuous |
| sex | As male or female which was later coded as 0=male, 1=female |
| Current smoking | 0=non-smokier, 1=current smoker |
| Current drinker | 0=non-drinker, 1=drinker. Or if we have grams of weekly alcohol intake, 0=non-drinker, 1=any grams of alcohol consumed |
| Physical activity | These were continuous variables in the datasets. We used the functional form of the physical activity variable and dissected this variable at the breakpoint. 0=low physical activity, 1=high physical activity |
