# Supplementary material for: Analysis of ancestry-specific polygenic risk score and diet composition in type 2 diabetes
Source: PLoS One. 2023 May 23;18(5):e0285827. doi: 10.1371/journal.pone.0285827 (PMC10204962; doi:10.1371/journal.pone.0285827)
Supplement: S3 Table — Abbreviations: NHLBI, National Heart, Lung, and Blood Institute; Care, Candidate Gene Association Resource; ARIC, Atherosclerosis Risk in Communities study [16], CARDIA, Coronary Artery Risk Development in Young Adults Study [17], CHS, Cardiovascular Heart Study [18], FHS, Framingham Heart Study Offspring and GENX 3 studies [19], MESA, Multi-Ethnic Study of Atherosclerosis Study [20], and Women’s Health Initiative study (WHI) [21]. (DOCX) [file pone.0285827.s003.docx]

**S3 Table. Characteristics of diet forms used by the seven NHLBI Care studies.**

| **Study** | **Diet Form** | **Items** | **Exam Year Used** |
| --- | --- | --- | --- |
| ARIC | FFQ | semi-quantitative 66-item | 1987-Exam 1 |
| CARDIA | Diet history | 102 questions | 1989-Exam 1 |
| CHS | Diet history | 96 questions | 1998-Exam 1 |
| FHS OFFSPRING | FFQ | semi-quantitative 88-items | 1983-Exam 3 |
| FHS GENX 3 | FFQ | semi-quantitative 88-items | 2002-Exam 2 |
| MESA | FFQ | semi-quantitative 153-items | 2010-Exam 5 |
| WHI | FFQ | semi-quantitative 145-items | 2005-Exam 1 |
