# Supplementary material for: Analysis of ancestry-specific polygenic risk score and diet composition in type 2 diabetes
Source: PLoS One. 2023 May 23;18(5):e0285827. doi: 10.1371/journal.pone.0285827 (PMC10204962; doi:10.1371/journal.pone.0285827)
Supplement: S4 Table — Bold indicates p values that were statistically significant at p < .05.*Bonferroni adjustment for multiple testing for dietary patterns (p = .05/2 = .0125). Macronutrient intakes were calculated as percent of total caloric intake. Type 2 diabetes status was regressed against each macronutrient using Generalized Estimating Equation model, adjusting for a covariate propensity score composed of age, sex, physical activity, current cigarette smoking status, current drinking status, carbohydrate, protein, and fat intake stratified by PRS tertiles to derive odds ratios and 95% confidence intervals. When a specific nutrient was the focus of interest, it was not included in the covariate propensity score. (DOCX) [file pone.0285827.s004.docx]

**S4 Table. Association between a polygenic risk score (PRS) and carbohydrate and protein intake with type 2 diabetes in European Americans adjusted for all covariates.**

|  | **Odds Ratio(95% Confidence Interval) P Value** | | | | | |  |
| --- | --- | --- | --- | --- | --- | --- | --- |
|  | **Lowest PRS Tertile** | **P Value** | **Second PRS Tertile** | **P Value** | | **Highest PRS Tertile** | **P Value** |
|  |  |  | **European Americans** |  | |  |  |
|  | (n=1172) |  | (n=2606) | |  | (n=2756) |  |
| **Carbohydrate (high vs. low)** |  |  |  | |  |  |  |
| Lowest tertile | 1.00(ref) |  | 1.00(ref) | |  | 1.00(ref) |  |
| Second tertile | 0.77(0.53-1.13) | .190 | 1.03(0.79-1.34) | | .845 | 0.97(0.74-1.27) | .826 |
| Highest tertile | 0.69(0.45-1.05) | .471 | 0.90(0.68-1.19) | | .453 | **0.68(0.51-0.92)** | **.012*** |
| **Protein (low vs. high)** |  |  |  | |  |  |  |
| Highest tertile | 1.00(ref) |  | 1.00(ref) | |  | 1.00(ref) |  |
| Second tertile | **0.64(0.43-0.95)** | **.027** | 0.94(0.71-1.24) | | .668 | **0.73(0.56-0.96)** | **.025** |
| Lowest tertile | **0.55(0.36-0.82)** | **.004*** | 0.81(0.61-1.08) | | .154 | **0.56(0.42-0.75)** | **.001*** |
| **Reverse of Above** |  |  |  |  | |  |  |
| **Carbohydrate (low vs. high)** |  |  |  |  | |  |  |
| Highest tertile | 1.00(ref) |  | 1.00(ref) |  | | 1.00(ref) |  |
| Second tertile | 1.12(0.73-1.72) | .598 | 1.14(0.86-1.52) | .356 | | **1.42(1.06-1.90)** | **.020** |
| Lowest tertile | 1.45(0.95-2.20) | .083 | 1.11(0.84-1.47) | .453 | | **1.46(1.09-1.96)** | **.012*** |
| **Protein (high vs. low)** |  |  |  |  | |  |  |
| Lowest tertile | 1.00(ref) |  | 1.00(ref) |  | | 1.00(ref) |  |
| Second tertile | 1.17(0.78-1.76) | .440 | 1.16(0.88-1.52) | .301 | | 1.31(0.97-1.78) | .077 |
| Highest tertile | **1.83(1.22-2.74)** | **.004*** | 1.23(0.93-1.63) | .154 | | **1.79(1.34-2.40)** | **.001*** |
