# Supplementary material for: Analysis of ancestry-specific polygenic risk score and diet composition in type 2 diabetes
Source: PLoS One. 2023 May 23;18(5):e0285827. doi: 10.1371/journal.pone.0285827 (PMC10204962; doi:10.1371/journal.pone.0285827)
Supplement: S6 Table — Abbreviations: PRS, polygenic risk score; HDL, high density cholesterol. General estimating equation model was constructed by regressing type 2 diabetes status (yes/no) against dichotomized values (high vs. normal) for all metabolic factors (waist circumference, body mass index, systolic blood pressure, etc.) to derive odds ratios and 95% confidence intervals. adjusted for a propensity score that consisted of age, sex, physical activity, current smoking, current drinking, and 10 principal components for population stratification. (DOCX) [file pone.0285827.s006.docx]

**S6 Table. Association of a polygenic risk score and metabolic factors with type 2 diabetes.**

|  |  | **Odds Ratio(95% Confidence Interval) P Value** | | |  |  |
| --- | --- | --- | --- | --- | --- | --- |
|  | **Lowest PRS Tertile** | **P Value** | **Second PRS Tertile** | **P Value** | **Highest PRS Tertile** | **P Value** |
|  |  | **European Americans** | | |  |  |
|  | (n=2,318) |  | (n=2,747) |  | (n=2,764) |  |
| Waist circumference | **2.06(1.40-3.02)** | **< .0001** | **1.62(1.23-2.13)** | **.001** | **1.37(1.03-1.83)** | **.031** |
| Body mass index | 1.20(0.98-1.47) | .080 | 1.11(0.96-1.29) | .157 | **1.32(1.12-1.55)** | **.001** |
| Systolic blood pressure | 1.33(0.94-1.87) | .108 | **1.33(1.04-1.71)** | **.025** | **1.52(1.16-1.99)** | **.002** |
| Hypertension medication | **3.36(2.39-4.72)** | **< .0001** | **1.76(1.38-2.25)** | **< .0001** | **2.10(1.63-2.69)** | **< .0001** |
| HDL cholesterol | 1.31(0.92-1.87) | .133 | 1.61(1.25-2.07) | **< .0001** | **1.63(1.25-2.13)** | **< .0001** |
| Triglycerides | **1.70(1.20-2.42)** | **.003** | **1.69(1.32-2.16)** | **< .0001** | **2.08(1.62-2.67)** | **< .0001** |
|  |  |  | **African Americans** |  |  |  |
|  | (n=518) |  | (n=517) |  | (n=517) |  |
| Waist circumference | 1.16(0.70-1.92) | .575 | 0.99(0.68-1.45) | .978 | **1.48(1.01-2.15)** | **.044** |
| Body mass index | **2.01(1.02-3.95)** | **.043** | 1.54(0.86-2.77) | .148 | **1.86(1.15-3.03)** | **.012** |
| Systolic blood pressure | 1.20(0.71-2.05) | .493 | 1.64(0.98-2.74) | .060 | 1.30(0.84-2.01) | .245 |
| Hypertension medication | **1.71(1.01-2.92)** | **.047** | **1.91(1.18-3.08)** | **.008** | 1.25(0.81-1.93) | .314 |
| HDL cholesterol | 1.70(1.00-2.89) | .051 | 0.98(0.59-1.64) | .939 | 1.32(0.83-2.11) | .239 |
| Triglycerides | **2.95(1.68-5.17)** | **< .0001** | 1.74(0.99-3.04) | .053 | **3.07(1.72-5.51)** | **< .0001** |
