# Supplementary material for: Analysis of ancestry-specific polygenic risk score and diet composition in type 2 diabetes
Source: PLoS One. 2023 May 23;18(5):e0285827. doi: 10.1371/journal.pone.0285827 (PMC10204962; doi:10.1371/journal.pone.0285827)
Supplement: Attachment — Submitted filename: PlosOne_T2DM_PRS.docx [file pone.0285827.s007.docx]

In short summary, this study found that individuals in the highest PRS tertile had higher T2DM risk in European Americans and African Americans. Protective effects were seen with combination of high carbohydrate, low protein intake and low PRS. African Americans benefited from increased physical activity by reduce harm of high protein diet.

Sections:

1. Disclosures:
   a. In financial disclosures, the authors mention a grant from the NLHBI to the first author. As this grant (K01) can include salaries and only NLHBI-sponsored studies were accessed, this should also be mentioned as conflict of interest. Otherwise, the authors should explain why this should not be the case.
2. Introduction:
   a. The authors should shortly state the clinical importance of their work at the end of the introduction and furthermore in the discussion and conclusion section.
3. Results:
   a. Bonferroni adjustments: The authors make a Bonferroni adjustment for both African American and European Americans, i.e. deduce a significance level of .025. I would like the authors to explain why a Bonferroni adjustment was not done for testing between different tertiles (e.g. in Table 3), as this is also a case of multiple testing. However, I am aware that this topic can be interpreted differentially. This would reduce the number of reported significant associations in the tables significantly.
   b. Tables 4 and 7 are quite hard to interpret as the information is somewhat ambiguous. The tables should be simplified or restructured to improve readability. Also, instructions of how to interpret the table should be given in the caption. For example, in Table 4, the authors compare tertiles within two groups, one with “High Carbohydrate and Low Protein Intake” and vice versa. Then, in the former group, the authors present data about Physical activity alone twice.
4. Conclusions:
   a. “African Americans can benefit from increased physical activity to decrease the harmful effects of a high protein diet despite having a deleterious burden of high-risk alleles.” (This is a literal quote from the abstract).
      This study only tested for associations, not causality, which should be stated in the paragraph listing the study’s limitations. The design of the study is a cross-sectional study that aims to examine the relationship between variables at one point in time, rather than to establish cause-and-effect relationships. Thus, for the next iteration of this work, the authors should rephrase this sentence such as “African Americans may benefit…” etc. as they did in the conclusions section in the main text.
   b. The authors should elaborate on the clinical significance of this paper, i.e. hypothesis generation for future RCTs or direct clinical implications.
5. General remarks:
   a. Length: The article is quite long and very technical. Perhaps, the authors can omit some of the presented text and tables and transfer them into the supplement.
   b. Captions: In general, captions should be optimized to give the reader more guidance of how the data has been interpreted.

Misspellings and Errors:

1. Line numbers suddenly stop at line 250 without apparent reason.
2. Line 65: should be "...that affects 37.3M (11.3%) people in the U.S."
3. Line 99: "was associated". Replace with "has been associated".
4. In the last sentence of the main text should be changed from “..harmful high protein diet…” to “ A high level of physical activity may be protective in African Americans with T2DM who consume a *harmfully* high protein diet…”.
