# Supplementary material for: Analysis of ancestry-specific polygenic risk score and diet composition in type 2 diabetes
Source: PLoS One. 2023 May 23;18(5):e0285827. doi: 10.1371/journal.pone.0285827 (PMC10204962; doi:10.1371/journal.pone.0285827)
Supplement: Attachment — Submitted filename: PlosOne_T2DM_PRS_REVISION_4-6-2023.docx [file pone.0285827.s008.docx]

April 7, 2023

Dear Editors and Reviewers,

We would like to thank both reviewers and the editorial office for their insightful and constructive comments. These comments have helped us improve and strengthen our article entitled, **Analysis of Ancestry-Specific Polygenic Risk Score and Diet Composition in Type 2 Diabetes.** We have modified the manuscript in addressing each comment. The marked version of the revised manuscript is attached as a supporting information file. Insertions in the text are in RED INK, whereas deletions are not shown to preserve clarity. The unmarked version is in plain text. We removed some of the tables and located them in a supplemental file. Because there are so many parts to this manuscript, we feel that by deleting information, our work might be less reproducible. Specific responses to critiques and suggestions are outlined in our point-by-point response below.

**Reviewers’ Comments:**

In short summary, this study found that individuals in the highest PRS tertile had higher T2DM risk in European Americans and African Americans. Protective effects were seen with combination of high carbohydrate, low protein intake and low PRS. African Americans benefited from increased physical activity by reduced harm of high protein diet.

| **Comments** | **Responses** |
| --- | --- |
| 1. Disclosures:  a. In financial disclosures, the authors mention a grant from the NLHBI to the first author. As this grant (K01) can include salaries and only NLHBI-sponsored studies were accessed, this should also be mentioned as conflict of interest. Otherwise, the authors should explain why this should not be the case. | NHLBI is a main sponsor of these types of studies, and our datasets were chosen from NHLBI studies in dbGaP. We also wanted to include studies that collected nutrition and physical activity data and other demographic, in addition to GWAS data and metabolic indices. |
| Introduction:  The authors should shortly state the clinical importance of their work at the end of the introduction and furthermore in the discussion and conclusion section. | Thank you for this suggestion. We made these additions to the abstract, introduction, discussion, and conclusion parts of the manuscript. |
| 2. Results:  a. Bonferroni adjustments: The authors make a Bonferroni adjustment for both African American and European Americans, i.e. deduce a significance level of .025. I would like the authors to explain why a Bonferroni adjustment was not done for testing between different tertiles (e.g. in Table 3), as this is also a case of multiple testing. However, I am aware that this topic can be interpreted differentially. This would reduce the number of reported significant associations in the tables significantly. | Thank you for this oversight. We made revisions to table 3 and table 7 to reflect Bonferroni corrections as the comparison of 4 tests: .05/4=.0125 as the cut-off instead of 2 tests. This p value is corrected for 4 tests for carbohydrate or protein intakes that includes the forward and reverse associations. We also reconfigured the tables and re-numbered the tables, making some of them supplemental tables so we can only have the main tables in the body of the manuscript. |
| b. Tables 4 and 7 are quite hard to interpret as the information is somewhat ambiguous. The tables should be simplified or restructured to improve readability. Also, instructions of how to interpret the table should be given in the caption. For example, in Table 4, the authors compare tertiles within two groups, one with “High Carbohydrate and Low Protein Intake” and vice versa. Then, in the former group, the authors present data about Physical activity alone twice. | Thank you for this suggestion. We made adjustments to these tables. We have reconstructed these tables to give information that is only needed to help interpretability to be clearer. |
| 3. Conclusions:  a. “African Americans can benefit from increased physical activity to decrease the harmful effects of a high protein diet despite having a deleterious burden of high-risk alleles.” (This is a literal quote from the abstract).  This study only tested for associations, not causality, which should be stated in the paragraph listing the study’s limitations. The design of the study is a cross-sectional study that aims to examine the relationship between variables at one point in time, rather than to establish cause-and-effect relationships. Thus, for the next iteration of this work, the authors should rephrase this sentence such as “African Americans may benefit…” etc. as they did in the conclusions section in the main text. | Thank you for your suggestion. We made revisions in the manuscript to reflect the cross-sectional limitations of the study design. In addition, we have rephrased the sentence pertaining to: “African Americans could benefit from increased physical activity to decrease the harmful effects of a high protein diet despite having a deleterious burden of high-risk alleles.” |
| b. The authors should elaborate on the clinical significance of this paper, i.e. hypothesis generation for future RCTs or direct clinical implications. | Thank you for your suggestions. We elaborated on the clinical significance of the associations we found. |
| 4. General remarks:  a. Length: The article is quite long and very technical. Perhaps, the authors can omit some of the presented text and tables and transfer them into the supplement. | Thank you for your suggestions. We retained the text related to the methods as vital information. We transferred several tables to the supplemental section of the paper. |
| b. Captions: In general, captions should be optimized to give the reader more guidance of how the data has been interpreted. | We added more explanation that interprets the results in the tables. |
| Misspellings and Errors:  1. Line numbers suddenly stop at line 250 without apparent reason.  2. Line 65: should be "...that affects 37.3M (11.3%) people in the U.S."  3. Line 99: "was associated". Replace with "has been associated".  1. In the last sentence of the main text should be changed from “..harmful high protein diet…” to “ A high level of physical activity may be protective in African Americans with T2DM who consume a harmfully high protein diet…”. | Thank you for all your suggestions. We have made all the changes as suggested. |
|  |  |
| Thank you for stating in your Funding Statement:  "This work is supported by a K01 grant from the National Heart, Lung, and Blood Institute grants: K01 HL127278 awarded to Dale Hardy, PhD." |  |
| Please provide an amended statement that declares *all* the funding or sources of support (whether external or internal to your organization) received during this study, as detailed online in our guide for authors at http://journals.plos.org/plosone/s/submit-now. ; Please also include the statement “There was no additional external funding received for this study.” in your updated Funding Statement.  Please include your amended Funding Statement within your cover letter. We will change the online submission form on your behalf. | Thank you for your recommendation. We have amended the funding statement as suggested. Our financial disclosure is below.  **Financial Disclosure**  This work is supported by a K01 grant from the National Heart, Lung, and Blood Institute (NHLBI) grants: K01 HL127278 awarded to Dale Hardy, PhD. There was no additional external funding received for this study. The NHLBI had no role in study design, data collection and analysis, decision to publish, or preparation of the manuscript. |
| Thank you for stating the following in your Competing Interests section:  "NO authors have competing interests"  Please complete your Competing Interests on the online submission form to state any Competing Interests. If you have no competing interests, please state ""The authors have declared that no competing interests exist."", as detailed online in our guide for authors at http://journals.plos.org/plosone/s/submit-now.  This information should be included in your cover letter; we will change the online submission form on your behalf. | Thank you for your suggestion. We have completed all requests as recommended. |
| 5. In your Data Availability statement, you have not specified where the minimal data set underlying the results described in your manuscript can be found. PLOS defines a study's minimal data set as the underlying data used to reach the conclusions drawn in the manuscript and any additional data required to replicate the reported study findings in their entirety. All PLOS journals require that the minimal data set be made fully available. For more information about our data policy, please see http://journals.plos.org/plosone/s/data-availability. | Thank you for pointing out this oversight. Dr. Hardy has converse with dbGaP. dbGaP pointed Dr. Hardy to the accession numbers for investigators to request the datasets. dbGaP data is not freely available data and is bounded by the dbGaP Data Use Agreement document. |
| Upon re-submitting your revised manuscript, please upload your study’s minimal underlying data set as either Supporting Information files or to a stable, public repository and include the relevant URLs, DOIs, or accession numbers within your revised cover letter. For a list of acceptable repositories, please see http://journals.plos.org/plosone/s/data-availability#loc-recommended-repositories. Any potentially identifying patient information must be fully anonymized. | Thank you for pointing out this oversight. We have provided information under the accession numbers in dbGaP, the curator and the repository of the data. dbGaP data is not freely available data. The data should be requested with the accession numbers included. |
| Important: If there are ethical or legal restrictions to sharing your data publicly, please explain these restrictions in detail. Please see our guidelines for more information on what we consider unacceptable restrictions to publicly sharing data: http://journals.plos.org/plosone/s/data-availability#loc-unacceptable-data-access-restrictions. Note that it is not acceptable for the authors to be the sole named individuals responsible for ensuring data access.  We will update your Data Availability statement to reflect the information you provide in your cover letter. | The data is from dbGaP and is not freely available without restrictions. Below is an excerpt from dbGaP data use agreement policy.  *--Under the National Institutes of Health (NIH) Genomic Data Sharing Policy, the Genomic Data User Code of Conduct sets forth principles for responsible management and use of large-scale genomic data and associated phenotypic data accessed through controlled access to NIH designated data repositories (e.g., the database of Genotypes and Phenotypes (dbGaP), repositories established as NIH Trusted Partners). Failure to abide by any term within this Code of Conduct may result in revocation of approved access to datasets obtained through these repositories.--* |
| PLOS requires an ORCID iD for the corresponding author in Editorial Manager on papers submitted after December 6th, 2016. Please ensure that you have an ORCID iD and that it is validated in Editorial Manager. To do this, go to ‘Update my Information’ (in the upper left-hand corner of the main menu), and click on the Fetch/Validate link next to the ORCID field. This will take you to the ORCID site and allow you to create a new iD or authenticate a pre-existing iD in Editorial Manager. Please see the following video for instructions on linking an ORCID iD to your Editorial Manager account: https://www.youtube.com/watch?v=_xcclfuvtxQ | Thank you for your suggestion. We have updated Dr. Hardy’s ORCID ID in Plos One’s Editorial Manager.  My ORCID ID: 0000-0001-8778-5661 |
| 7. We note that you have included the phrase “data not shown” in your manuscript. Unfortunately, this does not meet our data sharing requirements. PLOS does not permit references to inaccessible data. We require that authors provide all relevant data within the paper, Supporting Information files, or in an acceptable, public repository. Please add a citation to support this phrase or upload the data that corresponds with these findings to a stable repository (such as Figshare or Dryad) and provide and URLs, DOIs, or accession numbers that may be used to access these data. Or, if the data are not a core part of the research being presented in your study, we ask that you remove the phrase that refers to these data. | Thank you for this suggestion. We have revised the statement as recommended. |
| 8. Please include your full ethics statement in the ‘Methods’ section of your manuscript file. In your statement, please include the full name of the IRB or ethics committee who approved or waived your study, as well as whether or not you obtained informed written or verbal consent. If consent was waived for your study, please include this information in your statement as well. | Thank you for your suggestion. We have included this information in the METHODS section of the manuscript. All changes were made as suggested. We have added more explanations to support our conclusions. |
| **Additional Editor Comments:**  **Reviewer 1:**  In short summary, this study found that individuals in the highest PRS tertile had higher T2DM risk in European Americans and African Americans. Protective effects were seen with combination of high carbohydrate, low protein intake and low PRS. African Americans benefited from increased physical activity by reduce harm of high protein diet.  Sections:  1. Disclosures:  a. In financial disclosures, the authors mention a grant from the NLHBI to the first author. As this grant (K01) can include salaries and only NLHBI-sponsored studies were accessed, this should also be mentioned as conflict of interest. Otherwise, the authors should explain why this should not be the case. |  |
| 2. Introduction:  a. The authors should shortly state the clinical importance of their work at the end of the introduction and furthermore in the discussion and conclusion section. | Thank you for your suggestion. We have included this information in the different sections of the manuscript. |
| 3. Results:  a. Bonferroni adjustments: The authors make a Bonferroni adjustment for both African American and European Americans, i.e. deduce a significance level of .025. I would like the authors to explain why a Bonferroni adjustment was not done for testing between different tertiles (e.g. in Table 3), as this is also a case of multiple testing. However, I am aware that this topic can be interpreted differentially. This would reduce the number of reported significant associations in the tables significantly. | Thank you for your suggestion. We made the Bonferroni correction more stringent to include all carbohydrate or protein equations (4 each). We therefore changed Bonferroni correction from p-.025 to p=.0125. |
| b. Tables 4 and 7 are quite hard to interpret as the information is somewhat ambiguous. The tables should be simplified or restructured to improve readability. Also, instructions of how to interpret the table should be given in the caption. For example, in Table 4, the authors compare tertiles within two groups, one with “High Carbohydrate and Low Protein Intake” and vice versa. Then, in the former group, the authors present data about Physical activity alone twice. | Thank you for your suggestions. We have reconstructed Tables 4 and 7 and re-numbered Table 7 as Table 5 and showed only information pertaining to what we discussed in this portion of the manuscript. |
| 4. Conclusions:  a. “African Americans can benefit from increased physical activity to decrease the harmful effects of a high protein diet despite having a deleterious burden of high-risk alleles.” (This is a literal quote from the abstract).  This study only tested for associations, not causality, which should be stated in the paragraph listing the study’s limitations. The design of the study is a cross-sectional study that aims to examine the relationship between variables at one point in time, rather than to establish cause-and-effect relationships. Thus, for the next iteration of this work, the authors should rephrase this sentence such as “African Americans may benefit…” etc. as they did in the conclusions section in the main text. | Thank you for your suggestions. We have made all these changes to the manuscript as recommended. |
| b. The authors should elaborate on the clinical significance of this paper, i.e. hypothesis generation for future RCTs or direct clinical implications. | We have added parts on clinical significance in the INTRODUCTION, DISCUSSION AND CONCLUSONS of the manuscript. |
| General remarks:  a. Length: The article is quite long and very technical. Perhaps, the authors can omit some of the presented text and tables and transfer them into the supplement. | Thank you for your suggestion. We have transferred several tables to the Supplemental tables section. We agree the study is very technical and brings together many different areas of research (genetics, diet, physical activity, bioinformatics). However, we want to explain carefully to the readers, the methods and results of all the analyses because we think it is important for our results. |
| Captions: In general, captions should be optimized to give the reader more guidance of how the data has been interpreted. | We have added captions to the tables to make our analyses and results clearer to the readers so that the tables can stand-alone and are clear in understanding. |
| Misspellings and Errors:  1. Line numbers suddenly stop at line 250 without apparent reason.  2. Line 65: should be "...that affects 37.3M (11.3%) people in the U.S."  3. Line 99: "was associated". Replace with "has been associated".  4. In the last sentence of the main text should be changed from “..harmful high protein diet…” to “ A high level of physical activity may be protective in African Americans with T2DM who consume a harmfully high protein diet…”. | Thank you for your recommendations. We have made all the changes as suggested to the manuscript. |
| **Reviewer 2:**  The authors performed a retrospective study of the influence of a polygenic risk score (PRS), diet and physical activity on the occurrence of diabetes mellitus type 2 (T2DM). They showed in an analysis from many genetic studies that a PRS influenced the occurrence of DMT2. However, high carbohydrate and low protein intake were associated with reduced risk of T2DM.  This observational study contradicts the results of prospective interventional studies, probably due to bias: Since more than 15 years, low carbohydrate diet was led to improved glycaemic control in T2DM patients (also in randomized studies) [Nutr Metab (Lond). 2008; 5: 9.]. Therefore, national and international guidelines mainly recommended carbohydrate restriction in T2DM patients. (In the last years, there was a change towards the assessment of the quality of carbohydrates, but this is still a matter of debate). Consequently, all patients with T2DM in the last decades received recommendations to reduce carbohydrate intake and increase protein intake. Patients included in the genetic studies may also have received the same recommendation and therefore either (1) have changed their dietary habits or (2) at least report that they would have changed their dietary habits in interviews. This would have led to this carbohydrate “paradox” in this study. What do the authors think about this problematic bias? I encourage the authors to clearly state in the discussion that this is only a retrospective study with associations. | Thank you for your insightful comments. Concerning your statement of bias. We have addressed this comment in the Discussion section.  Our addition to the manuscript concerning this point is below:  Low carbohydrate and high protein diets for T2DM patients are currently being prescribed by physicians and other health professionals for optimal blood glucose control, weight loss, and reversal of other abnormal metabolic parameters [57]. Although some studies show promising results, the consensus in a systematic review and a meta-analysis show otherwise, that there is no difference in effect for low carbohydrate and high protein diets on blood glucose control in the absence of weight loss [58]. The low carbohydrate and high protein diets may not be superior to other dietary patterns, such as the Mediterranean diet or the vegetarian diet for improvement in high blood glucose [57,59]. Diets that promote more satiety and less energy consumed contribute to weight loss and better blood glucose control [60]. Conversely, other studies show concern with high protein intake as it relates to exacerbation of chronic kidney disease in patients with or without this pre-existing condition [61]. Combined healthy behaviors such as maintaining healthy body weight, consuming a healthy diet, frequent exercise, smoking abstinence and limited alcohol intake have been associated with 80% lower incident T2DM risk [62]. |
| I discourage the use of the word “protective” as the study has no longitudinal data included, therefore no “protective” behavioral factors can be derived from this data. | Thank you for this suggestion. We have re-worded these parts of the manuscript to use the word -lower- instead of -protective-. |
| Furthermore, the authors discuss the possibility of “ketogenic diet” with means a total cut of carbohydrates and clearly contradicts the results of this study. | Thank you for pointing out this oversight. We have revised this section of the manuscript to make it clearer. |
| Furthermore, the authors should describe the source data better: Were those cross-sectional or longitudinal data? (this should be already mentioned in the Methods section). How were patients included? Do the patients represent the same characteristics as the general population? Did patients receive compensation?  In general, the manuscript is well written, statistics and references are adequate. The manuscript is generally very long, the results section may be shortened. | Thank you for pointing out this oversight. We have described the study design in a few sections of the manuscript. This includes how patients were included. |
| Minor comments:  - The first half of the first paragraph at page 14 should be moved to the Methods section.  - The authors write that the ADA currently recommends a high carbohydrate, low protein diet with reference [9]. However, the reference [9] refers to the definition of T2DM, and does not recommend any dietary measure. In fact, this reference does not even contain the words “carbohydrate” or “protein”.  - The main endpoint (occurrence of T2DM) is not clear from the abstract.  - Specific steps and changed variables during data harmonization should be summarized in a supplemental table.  - Was multiple imputation performed for missing data?  [Note: HTML markup is below. Please do not edit.] | Thank you for these suggestions. We have updated these parts of the manuscript as suggested. |
| 3. Have the authors made all data underlying the findings in their manuscript fully available?  The PLOS Data policy requires authors to make all data underlying the findings described in their manuscript fully available without restriction, with rare exception (please refer to the Data Availability Statement in the manuscript PDF file). The data should be provided as part of the manuscript or its supporting information, or deposited to a public repository. For example, in addition to summary statistics, the data points behind means, medians and variance measures should be available. If there are restrictions on publicly sharing data—e.g. participant privacy or use of data from a third party—those must be specified.  Reviewer #1: No  Reviewer #2: Yes | The data is available in dbGaP under their respective accession numbers. This is reported at the bottom of the text and in the Editorial Manager. |
| 4. Is the manuscript presented in an intelligible fashion and written in standard English?  PLOS ONE does not copyedit accepted manuscripts, so the language in submitted articles must be clear, correct, and unambiguous. Any typographical or grammatical errors should be corrected at revision, so please note any specific errors here.  Reviewer #1: Yes  Reviewer #2: Yes | Thank you for your comments. |
| Sections:  1. Disclosures:  a. In financial disclosures, the authors mention a grant from the NLHBI to the first author. As this grant (K01) can include salaries and only NLHBI-sponsored studies were accessed, this should also be mentioned as conflict of interest. Otherwise, the authors should explain why this should not be the case. | Because of the nature of this study, we needed a dataset that contained different data: genetic (GWAS) data, nutrition data, physical activity data and covariate data. The NHLBI Care datasets that we used provided these data needed to implement this study. |
| 2. Introduction:  a. The authors should shortly state the clinical importance of their work at the end of the introduction and furthermore in the discussion and conclusion section. | Thank you for this suggestion. We have addressed this important information in these parts of the manuscript. |
| 3. Results:  a. Bonferroni adjustments: The authors make a Bonferroni adjustment for both African American and European Americans, i.e. deduce a significance level of .025. I would like the authors to explain why a Bonferroni adjustment was not done for testing between different tertiles (e.g. in Table 3), as this is also a case of multiple testing. However, I am aware that this topic can be interpreted differentially. This would reduce the number of reported significant associations in the tables significantly. | The information provided in the results show results from the highest tertile and the second tertile compared to the lowest tertile or the reverse for carbohydrate or protein intake. We tightened the Bonferroni correction cutoff to include all 4 results from carbohydrate or protein intake (forward or reverse). So instead of using Bonferroni of .05/2=.025, we used .05/4=.0125 as the cutoff. |
| b. Tables 4 and 7 are quite hard to interpret as the information is somewhat ambiguous. The tables should be simplified or restructured to improve readability. Also, instructions of how to interpret the table should be given in the caption. For example, in Table 4, the authors compare tertiles within two groups, one with “High Carbohydrate and Low Protein Intake” and vice versa. Then, in the former group, the authors present data about Physical activity alone twice. | Thank you for your suggestion. We cleaned up these tables and concentrated our results on the information we wanted to present. |
| 4. Conclusions:  a. “African Americans can benefit from increased physical activity to decrease the harmful effects of a high protein diet despite having a deleterious burden of high-risk alleles.” (This is a literal quote from the abstract). | Thank you for pointing out this flaw. We have re-worded this sentence to make it more unique. |
| b. The authors should elaborate on the clinical significance of this paper, i.e. hypothesis generation for future RCTs or direct clinical implications. | Thank you for pointing out this oversight. We have added verbiage about clinical significance in various parts of the paper. |
| 5. General remarks:  a. Length: The article is quite long and very technical. Perhaps, the authors can omit some of the presented text and tables and transfer them into the supplement. | Thank you for this insightful suggestion. We have rearranged the tables and made some of them supplemental tables. |
| b. Captions: In general, captions should be optimized to give the reader more guidance of how the data has been interpreted. | We have added captions to optimize guidance to the reader about the interpretation of the data. |
| Misspellings and Errors:  1. Line numbers suddenly stop at line 250 without apparent reason.  2. Line 65: should be "...that affects 37.3M (11.3%) people in the U.S."  3. Line 99: "was associated". Replace with "has been associated".  4. In the last sentence of the main text should be changed from “..harmful high protein diet…” to “ A high level of physical activity may be protective in African Americans with T2DM who consume a harmfully high protein diet…”. | Thank you for these suggestions. We have made revision to these areas of the manuscript to make it better. |
| Reviewer #2: The authors performed a retrospective study of the influence of a polygenic risk score (PRS), diet and physical activity on the occurrence of diabetes mellitus type 2 (T2DM). They showed in an analysis from many genetic studies that a PRS influenced the occurrence of DMT2. However, high carbohydrate and low protein intake were associated with reduced risk of T2DM.  This observational study contradicts the results of prospective interventional studies, probably due to bias: Since more than 15 years, low carbohydrate diet was led to improved glycaemic control in T2DM patients (also in randomized studies) [Nutr Metab (Lond). 2008; 5: 9.]. Therefore, national and international guidelines mainly recommended carbohydrate restriction in T2DM patients. (In the last years, there was a change towards the assessment of the quality of carbohydrates, but this is still a matter of debate). Consequently, all patients with T2DM in the last decades received recommendations to reduce carbohydrate intake and increase protein intake. Patients included in the genetic studies may also have received the same recommendation and therefore either (1) have changed their dietary habits or (2) at least report that they would have changed their dietary habits in interviews. This would have led to this carbohydrate “paradox” in this study. What do the authors think about this problematic bias? I encourage the authors to clearly state in the discussion that this is only a retrospective study with associations. I discourage the use of the word “protective” as the study has no longitudinal data included, therefore no “protective” behavioural factors can be derived from this data. Furthermore, the authors discuss the possibility of “ketogenic diet” with means a total cut of carbohydrates and clearly contradicts the results of this study.  Furthermore, the authors should describe the source data better: Were those cross-sectional or longitudinal data? (this should be already mentioned in the Methods section). How were patients included? Do the patients represent the same characteristics as the general population? Did patients receive compensation?  In general, the manuscript is well written, statistics and references are adequate. The manuscript is generally very long, the results section may be shortened.  Minor comments:  - The first half of the first paragraph at page 14 should be moved to the Methods section.  - The authors write that the ADA currently recommends a high carbohydrate, low protein diet with reference [9]. However, the reference [9] refers to the definition of T2DM, and does not recommend any dietary measure. In fact, this reference does not even contain the words “carbohydrate” or “protein”.  - The main endpoint (occurrence of T2DM) is not clear from the abstract. | In the Abstract and in the whole manuscript we have reported the results were on risk for T2DM. We have made changes to the manuscript regarding ADA recommendations. We have revised the sentence about -T2DM and Alzheimer’s disease- to make it clearer.  The other comments were already addressed in different sections of the manuscript. |
| - Was multiple imputation performed for missing data? | We performed multiple imputations for genotype data on the Michigan Imputation Server and for covariates in Stata. This is stated in the manuscript in the methods section. |
