# Supplementary material for: Analysis of ancestry-specific polygenic risk score and diet composition in type 2 diabetes
Source: PLoS One. 2023 May 23;18(5):e0285827. doi: 10.1371/journal.pone.0285827 (PMC10204962; doi:10.1371/journal.pone.0285827)
Supplement: Attachment — Submitted filename: Response to reviewers.docx [file pone.0285827.s009.docx]

April 27, 2023

Dear Editors and Reviewers,

We would like to thank both reviewers and the editorial office for their rigorous review and their investment in their time and resources in making this paper better. These comments have helped us improve and strengthen our paper entitled, **Analysis of Ancestry-Specific Polygenic Risk Score and Diet Composition in Type 2 Diabetes.** We have attended to the comments in this second review of the paper. The marked version of the revised manuscript is attached as a supporting information file. Insertions in the text are in RED INK, whereas deletions are not shown to preserve clarity. The unmarked version is in plain text. We have addressed the reviewer comments on a point-by-point response below. To our knowledge, we do not have any retracted published manuscripts referenced in our paper.

**Reviewers’ Comments:**

6. Review Comments to the Author

Please use the space provided to explain your answers to the questions above. You may also include additional comments for the author, including concerns about dual publication, research ethics, or publication ethics. (Please upload your review as an attachment if it exceeds 20,000 characters)

| **Comments** | **Responses** |
| --- | --- |
| Reviewer #1: This retrospective study found that individuals within a higher PRS percentile had increased risk of developing T2DM. Furthermore, there was an association between a high protein low carbohydrate diet and risk of developing T2DM.  In my opinion, my posed questions have been sufficiently addressed. Especially the newly added captions to the figures enhance readability. | Thank you for your comment. Your contribution was instrumental in improving the paper to make it better. |
| Reviewer #2: The authors made substantial improvements to the manuscript. However, I believe that the manuscript can still be optimized:  - As already mentioned in the first review, the main outcome has to be reported in the abstract. I therefore recommend to add this information to the Methods section of the manuscript. | Thank you for pointing out this oversight. We have added a sentence to address the main outcome as type 2 diabetes in the METHODS section of the ABSTRACT. |
| - There is still missing information about data imputation in the manuscript (imputation by mean? Or median? Multiple imputation?) | Thank you for your recommendation. We have added the missing information about data imputation under the sub-section Statistical analysis on page 8, lines 176-179. |
